# Culturing on Wharton's Jelly Extract Delays Mesenchymal Stem Cell Senescence through p53 and p16INK4a/pRb Pathways

**DOI:** 10.1371/journal.pone.0058314

**Published:** 2013-03-13

**Authors:** Haojie Hao, Guanghui Chen, Jiejie Liu, Dongdong Ti, Yali Zhao, Shenjun Xu, Xiaobing Fu, Weidong Han

**Affiliations:** 1 Institute of Basic Medicine Science, Chinese PLA General Hospital, Beijing, China; 2 Department of Cardiology, Chinese PLA General Hospital, Beijing, China; University of Medicine and Dentistry of New Jersey, United States of America

## Abstract

Mesenchymal stem cells (MSCs) hold great therapeutic potential. However, MSCs undergo replication senescence during the *in vitro* expansion process. Wharton's jelly from the human umbilical cord harbors a large number of MSCs. In this study, we hypothesized that Wharton's jelly would be beneficial for *in vitro* expansion of MSCs. Wharton's jelly extract (WJEs), which is mainly composed of extracellular matrix and cytokines, was prepared as coating substrate. Human MSCs were isolated and cultured on WJE-coated plates. Although the proliferation capacity of cells was not augmented by WJE in early phase culture, adynamic growth in late-phase culture was clearly reduced, suggesting that the replicative senescence of MSCs was efficiently slowed by WJE. This was confirmed by β-galactosidase staining and telomere length measurements of MSCs in late-phase culture. In addition, the decreased differentiation ability of MSCs after long-term culture was largely ameliorated by WJE. Reactive oxygen species (ROS), p53, and p16INK4a/pRb expression increased with passaging. Analysis at the molecular level revealed that WJE-based culture efficiently suppressed the enhancement of intracellular ROS, p53, and p16INK4a/pRb in MSCs. These data demonstrated that WJE provided an ideal microenvironment for MSCs culture expansion *in vitro* preserved MSC properties by delaying MSCs senescence, and allowed large numbers of MSCs to be obtained for basic research and clinical therapies.

## Introduction

Mesenchymal stem cells (MSCs) possess self-renewal capacity and have the ability to differentiate into mesoderm, endoderm, and even ectoderm lineages [1]. They cause only minimal immunoreactivity [2] and secrete bioactive factors with anti-inflammatory and immunomodulatory effects [3]. MSCs are thought to have strong clinical potential for regenerative medicine and immunoregulatory therapeutic applications. MSCs can be derived from various human tissues such as bone marrow, umbilical cord, and placenta [4], [5]. However, they are extremely rare in primary tissues, and *in vitro* expansion by passaging is needed to obtain sufficient cells for clinical purposes. Similar to many other somatic cells, MSCs have a limited lifespan *in vitro*. After a certain number of cell divisions, MSCs enter senescence and stop proliferating. This is morphologically characterized by an enlarged and flattened cell shape. Furthermore, studies show that human MSCs exhibit reduced differentiation potential *in vitro* [6], and replicative senescence of MSC preparations begins with the first passage [7]. These problems have hindered the expansion of MSCs for therapeutic uses, causing a major bottleneck in clinical applications [8].

The mechanisms underlying the replicative senescence of MSCs involve two major molecular pathways. The p53 pathway, which is important in the DNA damage response as well as regulation of cellular senescence, is a mediator of telomere-dependent senescence [9]. An alterative, telomere-independent pathway, typically activated by intracellular reactive oxygen species (ROS), is mainly mediated by accumulation of cell cycle inhibitor proteins such as p16INK4a and pRb [10]. Although the exact upstream events that trigger p53 and p16INK4a/pRb pathway activation during culture expansion of MSC remain unclear, the dramatic microenvironmental change from *in vivo* to *in vitro* growth is reported to be a major factor [11]. Once removed from the native matrix, MSCs rapidly lose important characteristics because of an inadequately supportive culturing microenvironment. With extensive passaging, continuous accumulation of ROS, DNA damage and telomere shortening ultimately trigger cellular senescence [12]. Therefore, establishing an optimized microenvironment for MSC culture expansion that preserves the properties of MSCs is imperative.

In general, MSCs naturally reside in a specialized niche or microenvironment that may be crucial for regulating MSC survival, self-renewal, and differentiation [13]. A major component of this niche is the extracellular matrix (ECM). Several studies have attempted to simulate the natural niche of MSCs using an ECM that closely resembles the physiological ECM and provides a microenvironment optimized to retain MSC properties. Denatured collagen type I is a major protein of the ECM that preserves adiposity-related markers and MSC functions following extensive *ex vivo* expansion [14]. However, collagen type I is only one component of the MSC microenvironment. Reconstituting the intricate and highly ordered nature of the niche using synthetic or purified components is difficult. Native ECM, which also dramatically promotes MSC proliferation and preserves MSC properties, comes from reconstituting human bone marrow cells *ex vivo* [15]. ECM prepared from cultured bone marrow MSCs (BM-MSCs) of young mice (3 month old) that can rejuvenate senescent MSCs [16]. Nonetheless, several limitations for using MSCs still remain, such as finding a cell source, collecting sufficient amounts, and the problem of continuous replicative senescence. Obtaining native ECM for MSCs culture-expansion is difficult. Therefore, new biomaterials that simulate the MSC niche and reconstitute the optimized microenvironment are needed for large-scale enrichment of MSCs.

Umbilical cord Wharton's jelly-derived MSCs (UC-MSCs) are reported to have a better proliferation and expansion rate than adult BM-MSCs [17]. Wharton's jelly, which is a component of the UC-MSC niche, provides a natural microenvironment that preserves MSC properties. Therefore, we hypothesized that Wharton's jelly would be beneficial for MSC culture expansion *in vitro* and would preserve MSCs properties. In this study, Wharton's jelly extract (WJE) was generated from human umbilical cords. The main components of WJE were ECM proteins such as collagen, fibronectin and cytokines such as insulin-like growth factor I (IGF- I), and basic fibroblast growth factor β (bFGF). A WJE-coated surface provided an ideal environment to delay the senescence of MSCs through the p53 and p16INK4a/pRb pathways. This method could be useful for large-scale expansion of highly functional MSCs for therapeutic use.

## Materials and Methods

### Acquisition of human umbilical cords

We obtained human umbilical cord (UC) samples after the delivery of normal-term babies with approval of the Ethics Committee of Chinese People's Liberation Army Hospital and obtained signed informed consent forms from the donors.

### Preparation of human umbilical Wharton's jelly extracts, scanning electron microscopy and mass spectra analysis

UCs were collected in a transfer medium of phosphate-buffered saline (PBS) and 50 IU heparin, and were maintained at 4°C until processing, which was within 24 h of collection. UCs were washed three times in PBS, and the umbilical veins were rinsed with PBS to remove contaminating red blood cells. UCs were cut into 1 cm segments, and UC arteries, veins and amnion were removed. The gelatinous tissue was excised, and minced into 0.5–1 mm^3^ pieces. Equal volumes of PBS were used to swell the tissue pieces with constant shaking at 4°C for 48 h, and the tissue pieces were subjected to 5 freeze-thaw cycles (−80°C to 37°C). Samples were ruptured by high-speed dispersion on ice for 10 min, and homogenized with a glass homogenizer on ice. Tissue homogenates were centrifuged at 10000×g for 30 min to remove debris. Supernatants that were WJE were collected and protein concentrations were determined by Bradford assay (Bio-Rad, Hercules, CA, USA). WJE was diluted to 1% (w/v) with PBS and used to coat plastic culture plates at 4°C overnight, after which excess liquid was discarded. WJE-coated plates were washed two times with PBS before use.

WJE-coated plates were washed twice with PBS, fixed with 2.5% glutaraldehyde at 4°C for 24 h, and washed twice with PBS. Plates were dehydrated in ascending concentrations of ethanol (from 70% to 100%), and vacuum freeze-dried. Plates were submitted to sputtering with gold–palladium and specimens were examined using a CSM-950 scanning electron microscope (SEM) (Carl-Zeiss, Co., Oberkochen, Germany).

For mass spectra analysis, WJE proteins that coated the plate were extracted using lysis-buffer containing 7 M urea, 2 M thiourea, 2% CHAPS, 50 mM dithiothreitol (DTT), and 40 mM Tris (pH 8.8), followed by sonication. WJE protein samples were separated by polyacrylamide gel electrophoresis and analyzed with by silver staining. Relevant gel bands were excised and subjected to in-gel digestion with trypsin (Promega, WI, USA) using reported protocols [18]. Samples were dried at room temperature,and 1 µL α-Cyano-4-hydroxycinnamic acid (HCCA, Sigma-Aldrich, St, Louis, Mo, USA) substrate solution (3 g/L, dissolved in 50% acetonitrile and 2% trifluoroacetic acid) was added. Samples were analyzed by matrix-assisted laser desorption ionization-time of flight-mass spectrometry (MALDI-TOF-MS) (Bruke, Germany). Peptide fingerprints were matched according to Swiss-Prot protein database using Mascot http://www.matrixscience.com to identify proteins.

### Isolation, culture, and proliferation assays of UC- and bone marrow-derived MSCs

Wharton's jelly from UCs was excised, minced into 0.5–1 mm^3^ pieces using scissors and scalpels, and washed twice with PBS. Cells were released by treatment with 0.1% collagenase type II (Sigma-Aldrich, St, Louis, Mo, USA) for 16 h at 37°C. After digestion, cells were filtered through a 100 µM cell strainer (BD Falcon, NJ, USA) to remove tissue debris. Cells were washed 3 times by diluting with 5 volumes PBS, and centrifuged.

Cells were suspended in MSC culture growth medium which was Dulbecco's modified Eagle's medium (DMEM) (Invitrogen Co., Carlsbad, USA) with 10% (v/v) fetal bovine serum (FBS). All cultures were incubated at 37°C with 5% CO_2_ in a humidified chamber and cells were passaged using 0.05% trypsin−0.02% EDTA (Invitrogen Co., Carlsbad, USA) when they reached 70% confluence.

Human bone marrow samples were acquired from donors with approval from the Ethics Committee of PLA Hospital and informed consent forms signed by donors. Samples were diluted with PBS and layered on percoll (1.077 g/ml) (Amersham Pharmacia, Biotech), and centrifuged at 400×g for 30 min. The buffy coat containing mononuclear cells was washed twice with PBS and plated in MSC culture growth medium. All cultures were passaged when they reached 70% confluence.

For population doubling (PD) evaluation, MSCs were plated at 2000 cells/cm^2^ in T75 culture flasks or WJE-coated flasks in MSC culture growth medium. After counting and passaging at 70% confluence, cells were replated at the same cell density until signs of replicative senescence were evident. The numbers of PDs were calculated using the formula log_2_ (D/D_0_), where D_0_ = number cells seeded, and D = number of cells counted at confluence.

For 3-(4,5-dimethylthiazol-2-yl)-2,5-diphenyltetrazoliumbromide (MTT) assay, cells were plated onto plastic or WJE-coated 96-well plates (5×10^3^ cells/well) in MSC culture growth medium, and assays were performed from 1 to 6 days after plating. Growth medium containing 0.25 mg/ml MTT (Sigma-Aldrich, St, Louis, Mo, USA) was added to each well, and cells were incubated at 37°C for 20 min. Medium was replaced with 0.2 ml DMSO per well and MTT reduction was determined by measuring the optical density (OD) at 540 nm of DMSO extracts using DMSO as a blank control.

### Phenotype and differentiation features of MSCs

MSCs Phenotypes were determined using flow cytometry. UC-MSCs or bone marrow-MSCs (BM-MSCs) were detached using 0.05% trypsin−0.02% EDTA, resuspended in PBS containing 1% bovine serum albumin fraction V (BSA; Sigma-Aldrich) and divided into aliquots of 3×10^5^ cells. Cells were incubated for 30 min at 4°C with CD34-fluorescein isotiocyanate (FITC), CD45-phycoerythrin (PE), CD73-FITC, CD90-PE, CD105-allophycocyanin (APC), HLA-DR-APC, and isotypematched IgG-FITC, IgG-PE, IgG-APC control antibodies (BD Biosciences, USA). Analysis used a FACScan (BD, USA) for at least 10,000 events using CellQuest software (BD, USA).

Osteogenic and adipogenic differentiation was induced as previously described [19]. Adipogenic differentiation was assessed by incubating the cells in MSC culture growth media supplemented with 5 µg/mL insulin, 50 µM indomethacin, 1 µM dexamethasone, and 0.5 µM 3-isobutyl-1-methylxanthine. Medium was changed twice per week for 3 weeks. Cells were fixed with 10% formalin for 20 min and stained with 0.5% Oil Red O (all from Sigma-Aldrich, St, Louis, Mo, USA) in methanol for 20 min. To quantify Oil Red O, stained oil droplet were extracted by isopropyl alcohol and OD was measured at 520 nm.

Osteogenic differentiation was assessed by incubating cells in MSC culture growth media supplemented with 0.1 µM dexamethasone, 10 µM, β-glycero-phosphate, and 50 µM ascorbate. Medium was change twice per week for 3 weeks. Cells were fixed with 10% formalin for 20 min, and stained with alizarin red pH 4.1 for 20 min at 37°C. To quantify alizarin red, deposition was extracted by 10% (w/v) cetylpyridinium chloride (all from Sigma-Aldrich, St, Louis, Mo, USA) in 10 mM sodium phosphate (pH 7.0) for 1 h and the OD of the solution was measured at 560 nm.

### Senescence-associated β-galactosidase staining

Cells cultured on plates were washed 3 times with PBS and fixed with 3% paraformaldehyde for 15 min at room temperature. After washing 3 times with PBS, cells were incubated with a freshly prepared senescence-associated β-galactosidase (SA-β-gal) staining solution (Chemicon, USA) for 16 h at 37°C. Under light microscopy, the number of blue cells (SA-β-gal positive cells) out of at least 500 cells in 10 randomly chosen fields was used to calculate the percentage of senescent cells.

### Relative telomere length using quantitative real-time PCR

Relative telomere length was determined using quantitative real-time PCR assay as previously described [20]. Genomic DNA was extracted from MSCs and 293T cells using a DNA extraction kit (Takara, Japan). Relative telomere lengths were measured using SYBR green real-time quantitative PCR amplification of telomere repeats (T) and single-copy gene 36B4 (S). The 36B4 gene was used to normalize sample variations in DNA amount. T and S standard curves (Ct vs. log quantity) were generated using serially diluted of DNA (3.2 ng to 0.1 ng) from telomerase-positive 293T cells. Ct values in experimental samples were determined by semi-log amplification plots and standard curves were used to determine the quantity of telomere repeats. Relative telomere length was estimated as the T quantity/S quantity (T/S) ratio. Telomere and 36B4 PCR reactions were run on separate plates, and a standard curve was included with each run to allow relative quantification between samples (0.8 ng per sample). Primer sequences were: telomere 1 (Tel 1) 5′-GGTTTTTGAGGGTGAGGGTGAGGGTGAGGGTGAGGGT-3′ and (Tel 2) 5′-TCCCGACTATCCCTATCCCTATCCCTATCCCTATCCCTA-3′; 36B4 u 5′-CAGCAAGTGGGAAGGTGTAATCC-3′ and 36B4 d 5′-CCCATTCTATCATCAACGGGTACAA-3′. Reagents in PCR reactions were 25 µl 2×SYBR Green PCR Master Mix (Takara, Japan) with final primer concentrations: Tel 1, 270 nM; Tel 2, 900 nM; 36B4u, 300 nM; and 36B4d, 500 nM. Cycling were 95°C for 10 min, followed by 40 cycles of 95°C for 15 seconds, then 54°C for 2 min for telomere PCR; or 40 cycles of 95°C for 15 seconds then 58°C for 60 seconds for 36B4 PCR.

### Reactive oxygen species detection and immunoblotting assay

The intracellular generation of ROS was measured using 2′,7′-dichlorodihydrofluorescein diacetate (DCFH-DA, Molecular Probes, USA). After removing the medium, cells were washed three times with PBS. DCFH-DA diluted to a final concentration of 10 mM with L-DMEM was added to the growth medium, followed by incubation at 37°C for 15 min. Cells were washed once with PBS, and resuspend in PBS, then kept on ice for immediate detection by FACScan (BD, USA), with excitation at 488 and emission at 522 nm. The increase in value compared to the control indicated an increase in intracellular ROS.

Immunoblotting was performed as previously described [21]. Antibodies were against p53, p16INK4a, pRb, and β-actin (all from Santa Cruz, USA). Protein intensities were quantitatively measured using Quantity One (Bio-Rad).

### Statistical analysis

All data from quantitative experiments are presented as mean ± standard deviation (SD). All data represent an average of at least triplicate samples. Statistical analyses were done by Student's *t*-test for paired samples. Statistical significance was accepted for *P* values of < 0.05.

## Results

### Characteristics of WJE

**Figure 1:**
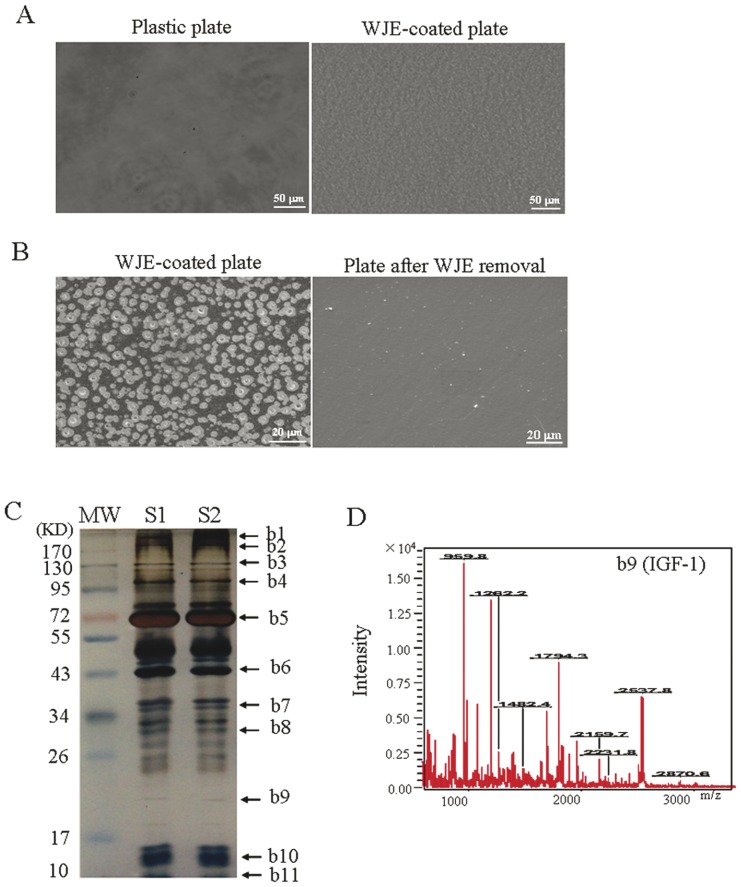
Characteristics of Wharton's Jelly extracts (WJE). (A) Micrographs from phase contrast microscopy of WJE-coated plates. Scale bars, 50 µm. (B) Scanning electron microscope images of WJE-coated plates. Scale bars, 20 µm. (C) Protein removed from WJE-coated plates analyzed by electrophoresis. (D) Mass spectra of a 21 kDa protein band by in-gel digestion with porcine trypsin (representative image). MW, molecular weight. S, sample. b, band.

Under phase contrast microscopy, a homogeneous distribution of a granular coating surface was observed on the WJE-coated plates (Fig. 1A). SEM revealed that the WJE-coated plates had spheres of diameter of about 2–5 µm (Fig. 1B). WJE was removed from plates and analyzed by polyacrylamide gel electrophoresis. WJE from two samples had similar sets of bands (Fig. 1C), indicating that different batches of WJE preparations were consistent. Sample bands were cut out and digested in-gel with trypsin for analysis by MALDF-TOF-MS. Of 11 bands, 8 were verified by comparison to their theoretical masses (Table 1). These results confirmed that the WJE contained collagen α-1(I), fibronectin and other ECM components, as well as cytokines such as IGF-1 and bFGF.

**Table 1:** Identified proteins extracted from WJE by MALDI-TOF-MS.

Number	Accession	Protein name	MW	PI	Mowse score
b1	P02751	Fibronectin	262624	5.46	908
b2	A4D0S4	LAMB4_HUMAN	193540	5.93	1020
b3	P02452	Collagen alpha-1(I)	138941	5.60	709
b4	P12110	CO6A2	108579	5.85	817
b5	P35475	IDUA	72670	9.25	588
b8	O15120	PLCB	30914	9.21	434
b9	P05019	IGF-1	21841	9.78	178
b11	P09038	bFGF	11670	11.71	88

Number, band number. MW, molecular weight (Dalton).

### Morphology, growth kinetics, and cell surface characteristics of long-term cultured UC-MSCs on WJE-coated plates

**Figure 2:**
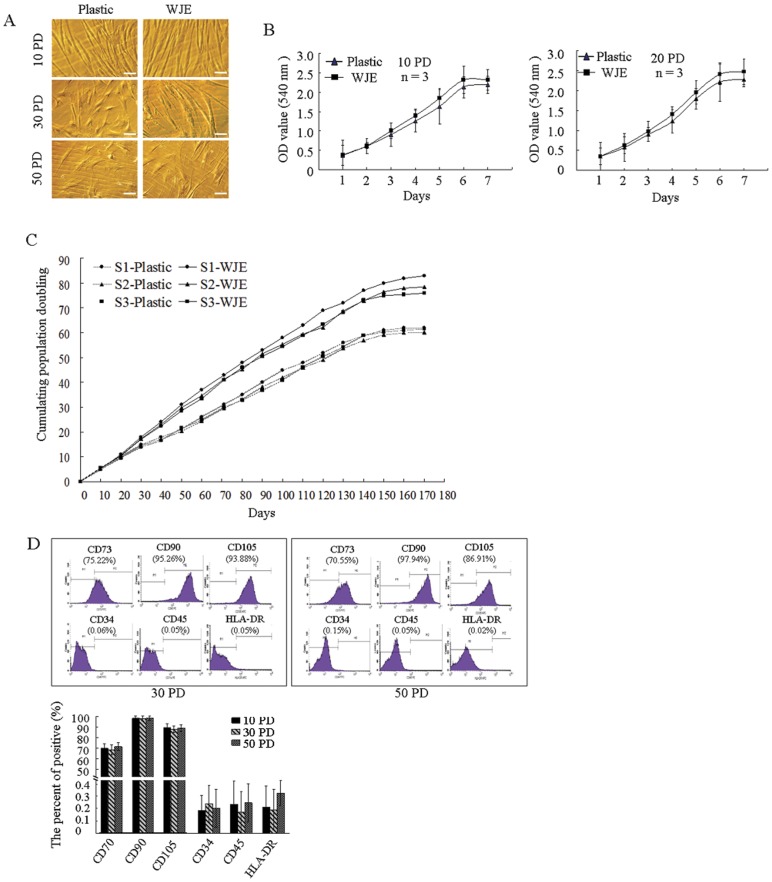
Characteristics of long-term cultured UC-MSCs on WJE-coated plates. (A) Morphological changes of long-term cultured UC-MSCs on WJE-coated plates by microscopy. Scale bars, 20 µm. (B) Proliferation ability of UC-MSCs cultured on WJE-coated plates by MTT assay. (C) Growth curve of long-term cultured UC-MSCs on WJE-coated plates. (D) Flow cytometric analysis of a representative example of UC-MSC (top). Histograms of a representative UC-MSCs culture on WJE-coated plate stained for lineage negative, and positive cell surface markers. Data are mean ± SD (n = 3) (*P>0.05).

Since MSCs underwent the typical Hayflick phenomenon of cellular senescence with decreasing proliferation and cell morphological changes, we monitored morphological changes in UC-MSCs. In the early-phase (10 PD) of culturing, UC-MSCs on both uncoated and WJE-coated plates were observed as slender, spindle-shaped, and fibroblast-like in morphology (Fig. 2A). In the middle-phase (30 PD) of culturing, cells on uncoated plates began to change to a flattened and enlarged phenotype, while cells on the WJE-coated plates remained fibroblast-like. In the late-phase (50 PD) of culturing, cells on uncoated plates exhibited a flat and hypertrophic phenotype, while most cells on WJE-coated plates remained spindle-shaped. These results demonstrated that the morphological changes associated with MSC senescence were mediated by WJE in middle- and late-phase cultures.

MTT assays were performed to compare the proliferation of UC-MSCs cultured on uncoated and WJE-coated plates. UC-MSCs were obtained from three donors, but no significant difference was observed between cells on uncoated or WJE-coated plates at 10 PD or 20 PD (Fig. 2B) (*P*<0.05). These results demonstrated that WJE-coating did not alter UC-MSC proliferation.

To investigate how WJE-coating affected the long-term growth kinetics of cells, we measured cumulative PDs of UC-MSCs cultured on uncoated and WJE-coated plates. The results from three samples on WJE-coated plates showed that the proliferative life span of UC-MSCs ended just before cell cultures reached 83, 78, and 76 PD. UC-MSCs plated on uncoated plates achieved only 62, 61, and 60 PD. Cells on uncoated plates had a similar regular growth rate to cells on WJE-coated plates before 15 PD. Post 30 PD, the proliferation of UC-MSCs cultured on uncoated plates appeared to gradually decrease. However, the proliferation of UC-MSCs cultured on WJE-coated plates remained the same until the end of culturing (Fig. 2C). The adynamic growth in late-phase cultures was clearly reduced by WJE, suggesting that the replicative senescence of MSCs was delayed efficiently by WJE.

Surface markers of UC-MSCs during long-term culture were characterized using FACS analysis. All 3 analyzed samples of UC-MSCs cultured on uncoated and WJE-coated plates at 10, 30, and 50 PD were positive for CD73, CD90, and CD105, but negative for CD34, CD45, and HLA-DR (Fig. 2D). No significant differences were observed between cells in early or late culture phase (*P*>0.05), consistent with other studies [22]. These results indicated that the surface markers of UC-MSCs were not affected by WJE.

### SA-β-gal activity and differentiation potential of UC-MSCs cultured on WJE-coated plates

**Figure 3:**
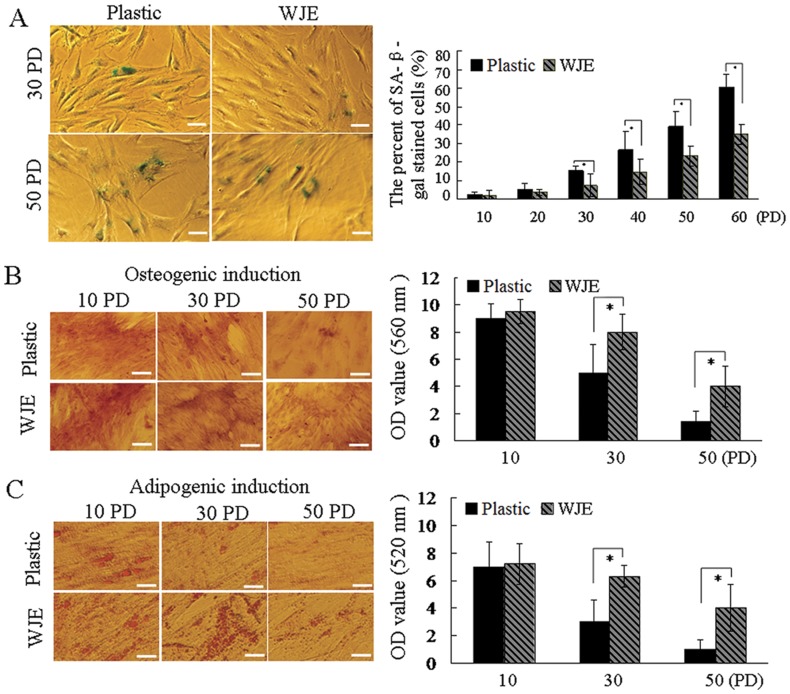
Senescence associated-β-galactosidase (SA-β-gal) activity and differentiation potential of UC-MSCs cultured on WJE-coated plates. (A) SA-β-gal staining of UC-MSCs cultured on uncoated and WJE-coated plates. Left panel, representative image of SA-β-gal staining, right panel, positive SA-β-gal staining. (B) Osteogenic differentiation of UC-MSCs. Representative image showing alizarin red staining (left). Histograms show staining ratios (right). (C) Adipogenic differentiation of UC-MSCs. Representative image showing Oil Red O staining (left). Histograms show staining ratios (right). Scale bars 20 µm. Data are mean ± S.E. (n = 3). (*P<0.05).

To further investigate the appearance of senescence, SA-β-gal activity of the cultured cells was analyzed. Before 20 PD, UC-MSCs cultured on uncoated or WJE-coated plates had limited SA-β-gal staining. All three samples of UC-MSCs cultured on uncoated plates showed a significant increase in the percentage of SA-β-gal-positive staining with passaging, while the proportion of senescent UC-MSCs cultured on WJE-coated plates increased slowly (Fig. 3A). These results showed that the replicative senescence of MSCs was efficiently mediated by WJE (*P*<0.05, n = 3). As early as 10 PD, UC-MSCs had a low SA-β-gal staining rate of only 1.9–2.2% positive cells. At 20 PD, the rate of SA-β-gal staining increased slightly to about 5%. This indicated that the replicative senescence of UC-MSCs was a continuous process from the early passage onwards, consistent with other reports [23]. These results are not in agreement with Terai [24]. That study did not detect SA-β-gal activity in MSCs in growth phase on day 59. We presume that the different results were because of culture environment, including medium composition. This further confirmed the importance of the environment.

To assess the differentiation potential of UC-MSCs, cells were cultured in osteogenic- and adipogenic-differentiation media. Alizarin red stain was used to detect precipitated calcium salt, which reflects osteogenic differentiation. The data from a visual assessment demonstrated that UC-MSCs cultured on uncoated plates showed lower amounts of alizarin red staining compared to cells on WJE-coated plates (Fig. 3B, left) at 30 PD and 50 PD. These results revealed that WJE increased the osteogenic differentiation ability of UC-MSCs that was reduced after long-term culture (*P*<0.05, n = 3) (Fig. 3B, right).

The adipogenic differentiation potential of UC-MSC was assessed by Oil Red O staining. Based on visual assessment of the extent Oil Red O-positive lipid inclusions in cells, the adipogenic differentiation potential of UC-MSCs on uncoated plates decreased with passaging compared to the WJE-coated group. In contrast, the adipogenic differentiation of UC-MSCs on WJE-coated plates was high (Fig. 3C). Quantification of Oil Red O staining indicated that WJE might ameliorate the decrease in adipogenic differentiation of UC-MSCs (*P*<0.05, n = 3) (Fig. 3C, right).

### Telomere length of UC-MSCs culture on WJE-coated plates

**Figure 4:**
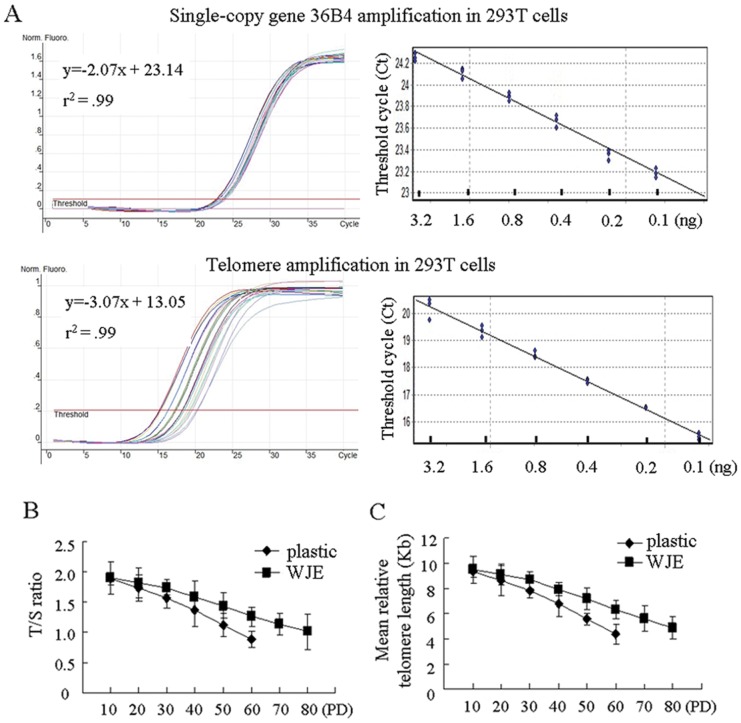
Relative telomere length expressed as T/S ratios. (A) Standard curve for T and S was from serial dilutions of DNA (3.20 ng to 0.10 ng) from reference 293T cells. (B) T/S ratios were plotted against passage number to show UC-MSCs distribution. (C) Mean relative telomere lengths of UC-MSCs. S, single-copy gene 36B4 amplification; T, telomere amplification.

Telomere erosion is considered to be a main cause of replicative senescence. Therefore, we determined the telomere length of UC-MSCs using real-time PCR. We used 293T cells as a positive control because they contain 5.0 kb of telomere. A standard curve of telomeres and the single-copy gene 36B4 in 293T cells showed that r^2^ values were consistently ≥ 0.99 (Fig. 4A). We applied a conversion factor [19] to convert T/S ratios to a corresponding mean telomere length. Telomere length shortened progressively in all three UC-MSCs samples with passaging (Fig. 4B, C). The mean of telomere lengths UC-MSCs on the uncoated plates were approximately 9.4, 7.84, and 5.6 kb at 10, 30, and 50 PD, while UC-MSCs on WJE-coated plates had telomeres of 9.5, 8.7, and 7.2 kb. The telomere length shortened from 9.4 kb to 4.4 kb on average, or 0.1 kb/PD when UC-MSCs were passaged on uncoated plates. However, when UC-MSCs were cultured on WJE-coated plates, the rate of telomere length shortening was 0.04 kb/PD, with an average of 0.077 kb/PD before 30 PD. These results indicated that shortening of the average telomere lengths of UC-MSCs could be slowed by WJE coating.

### WJE-coated also delays senescence of BM-MSCs

**Figure 5:**
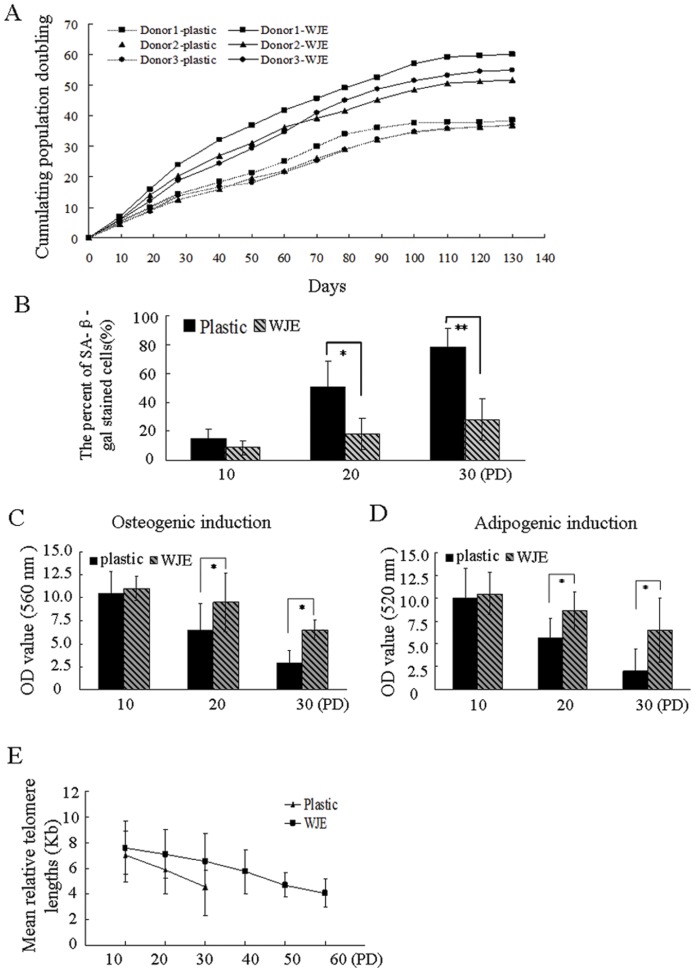
Delayed senescence of BM-MSCs cultured on WJE-coated plastes. (A) Long-term growth curves of BM-MSCs cultured on WJE-coated plates. (B) Positive SA-β-gal staining of BM-MSCs cultured on WJE-coated plates. (C) Osteogenic differentiation of BM-MSCs cultured on WJE-coated plates. (D) Adipogenic differentiation ability of BM-MSCs cultured on WJE-coated plates. (E) Mean telomere lengths of BM-MSCs determined by real-time PCR. Data are mean ± S.E (n = 3) (*P<0.05, **P<0.01).

We investigated the effect of BM-MSCs culturing on WJE-coated plates. BM-MSCs were obtained from three donors. BM-MSCs cultured on WJE-coated plates increased significantly and cumulatively starting at 8–12 PD (Fig. 5A). Furthermore, for BM-MSCs plated on WJE-coated plates, SA-β-gal positive cells were effectively reduced until 30 PD,then positive cells gradually increased (Fig.5B) (*P*<0.01, n = 3). BM-MSCs cultured on WJE-coated plates expanded to 52–60 PD, while cells grown on uncoated plates expanded to only 36–38 PD. Comparing their osteogenic- and adipogenic-differentiation potential at 20 PD and 30 PD, BM-MSCs cultured on WJE-coated plates had significantly increased potential compared to cells plated on uncoated plates (*P*<0.05) (Fig. 5C, D). These results demonstrated that the decreased differentiation ability of BM-MSCs after long-term culture could be ameliorated by WJE.

Measurement of mean telomere length by real-time PCR revealed that the average telomere length of BM-MSCs culture on uncoated plates ranged from 7.03 kb at 10 PD to 4.53 kb at 30 PD (on average, 0.125 kb/PD). In contrast, in BM-MSCs cultured on WJE-coated plates, the telomere was averaged 7.59 kb at 10 PD to 4.04 kb at 60 PD (on average 0.071 kb/PD). Although the decrease in telomere length of BM-MSCs cultured on WJE-coated plates declined more slowly than cells on uncoated plates, no significant difference was seen between groups (Fig. 5E). In both BM-MSCs and UC-MSCs cultured on WJE-coated plates, adynamic growth in late-phase culture was clearly reduced, suggesting that the replicative senescence of BM-MSCs was efficiently slowed by WJE.

### Culturing on WJE inhibits the accumulation of ROS and expression of p53, p16INK4a/pRb in MSCs

**Figure 6:**
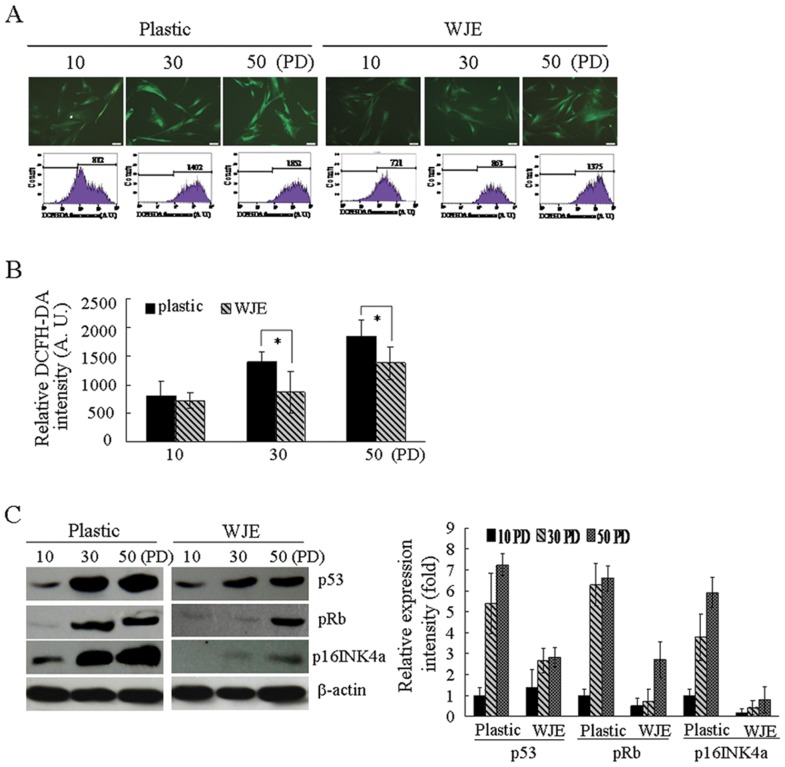
Suppression of intracellular ROS, p53, and p16INK4a/pRb of UC-MSCs cultured on WJE-coated plates. (A) Levels of intracellular ROS in UC-MSCs cultured on uncoated or WJE-coated plates measured by DCFH-DA (top) staining and confirmed by FACS analysis (bottom). (B) Histograms show relative DCFH-DA intensity. (C) Suppression of p53, p16INK4a/pRb expression in UC-MSCs cultured on WJE-coated plates at 30 PD or 50 PD compared with 10 PD. Histograms show quantitative measurements. Data are mean ± S.E. (n = 3) (*P<0.05).

Since ROS are well-known inducers of cellular senescence, we evaluated the level of intracellular ROS in UC-MSCs cultured on both uncoated and WJE-coated plates (Fig. 6A). ROS increased from early to late UC-MSCs passage, both for cells cultured on uncoated or WJE-coated plates, for all 3 samples. Compared to the uncoated group, the level of ROS in the group of UC-MSCs cultured on WJE-coated plates decreased slightly at 10 PD. However, a significant decrease appeared from 30 PD to 50 PD (Fig. 6B) (*P*<0.05, n = 3). These data showed that the ROS levels of UC-MSCs cultured on WJE-coated plates were lower than in cells grown on uncoated plates.

The p53 and p16INK4a/pRb pathways are important for triggering the MSC senescence process. We measured the expression of p53 and p16INK4a/pRb using Immunoblotting for different phases (Fig. 6C). Expression of p53, p16INK4a, and pRb increased gradually with cell passage. The expression of p53, p16INK4a, and pRb in UC-MSCs cultured on WJE-coated plates was decreased at 30 PD, 50 PD compared to cells cultured on uncoated plates (Fig. 6C). Specifically, we observed that p16INK4a expression had a greater dynamic range of several-fold changes. Fold differences in expression between cells on WJE-coated and uncoated plates were 5.5 at 10 PD, 8.8 at 30 PD, and 7.3 at 50 PD. Our data were consistent with studies reporting that the p53 and p16INK4a/pRb pathways converge on either or both of two pathways that reduce MSC senescence [25], [26]. We concluded that the WJE-coated surface provides an ideal environment that efficiently suppresses p53 and p16INK4a/pRb expression in UC-MSCs to delay replicative senescence of MSCs.

## Discussion

Although MSCs are believed to have promising therapeutic potentiality for regenerative medicine [27], they still have challenges to clinical application. One major challenge is replication senescence, which is inevitable during the *in vitro* expansion process. We developed a new strategy for MSC culture expansion *in vitro*. Wharton's jelly was extracted from the natural UC MSC environment of human umbilical cords. The WJE was used to coat the surface of culture plates to reconstruct the *in vivo* microenvironment of MSCs for the purpose of delaying MSC replication senescence *in vitro*. Compared to cells cultured on uncoated plastic plates, MSCs cultured on WJE-coated plates showed efficiently retarded replicative senescence.

The stem cell environment changes extensively during the lifetime of stem cells and this might be important for the external control of stem cell aging [28]. A previous study found that defects in the replication of senescence MSCs were completely restored when MSCs were exposed to an extracellular matrix environment generated by MSCs from young animals. This improvement diminished when cells from either young or old mice were cultured on an environment reconstituted from MSCs from old animals [16]. In addition, the aged phenotype of adult stem cells such as hematopoietic stem cells was reversed by exposing them to a young stem cell environment [29]. The precise mechanism of the interaction of stem cells with their local environment is largely unknown. However, the fate of MSCs is controlled by their interactions with their tissue-specific environment or niche, which consists of ECM components and soluble factors [30].

Wharton's jelly is a component of the UC-MSC *in vivo* environment that maintains the distinct features of UC-MSCs such as high CFU-F capability, high proliferative potential, short population doubling times and long life [20], [31]. Previous studies demonstrated that Wharton's jelly contains growth factors such as insulin-like growth factor I (IGF-1), bFGF, transforming growth factor β (TGF β), platelet-derived growth factor (P-DGF), epidermal growth factor(EGF)and ECM proteins [32]. These growth factors and ECM proteins constitute a microenvironment that not only provides structural and organizational guidance but also defines and maintains cellular phenotypes as well as driving decisions about cell fate [33], [34]. In particular, IGF-1 is considered to mediate the conservation of longevity and rejuvenate age-dependent stem cell defects [35]. In this study, we extracted WJE by physically breaking down Wharton's jelly. To acquire all components of Wharton's jelly, we used to repeated tissue freeze-thawing, homogenization, and high-speed centrifugation at low temperature to make a suspension of Wharton's jelly. WJE contained the main components of Wharton's jelly including ECM proteins such as collagen and fibronectin, and cytokines such as IGF-1 and bFGF. Furthermore, our results showed that replicative senescence of MSCs significantly delayed WJE, indicating that the WJE-coated plates were a good strategy for mimicking the MSC microenvironment for MSC culture expansion *in vitro*.

MSC senescence is considered to be associated with progressive shortening of telomere length and continuous accumulation of intracellular ROS [36], [37]. In the absence of telomerase, telomeres shorten in each round of cell division. When telomeres reach a critical length, they cannot maintain the proper structure of the chromosomal ends. The unprotected chromosomal ends activate DNA damage responses through signaling proteins such as p53, inducing replicative senescence [38], [39]. In BM-MSCs, the average telomere length is 9.17–10.4 kb in early passage cells and 7.1–8.7 kb in late-passage cells [40], [41]. Our results also showed that the senescence of MSCs accompanied progressive telomere shortening.

Increasing evidence indicates that the continuous accumulation of intracellular ROS is another major initiating factor of replicative senescence [42]. Furthermore, high levels of ROS are associated with the loss of stem cell self-renewal an increase in differentiation potential, and apoptosis in adult stem cells [43]. Our studies also showed that ROS levels were elevated increasing UC-MSCs senescence, and this trend can be inhibited by WJE.

Although the microenvironment controls MSC fate, replicative senescence is ultimately regulated by intrinsic stress signaling pathways. In MSCs, senescence is regulated in a telomere-dependent manner; growth cessation is primarily triggered by signals driven by p53 [44]. In many cases, telomere erosion and/or DNA damage also engage a p16INK4a/pRb checkpoint that triggers a permanent senescent state. However, the p53 and p16INK4a/pRb pathways appear to converge on either or both of two pathways that establish and maintain senescence or growth arrest [45]. A recent study showed that p16INK4a was closely associated with the senescence of MSCs. Clearance of the p16INK4a gene could delay the cellular aging process, and delay aging-associated disorders [46]. Similar to the p16INK4a-dependent mechanism of irreversible senescence, induction of p16INK4a but not p21 is responsible for premature senescence in MSCs [47]. In our results, we observed that the expression of both p53 and p16INK4a was enhanced in middle-phase and late-phase MSC cultures *in vitro*. The increasing expression of p53 and p16INK4a was significantly suppressed by WJE. These results suggested that WJE might delay MSCs replication senescence by inhibiting expression of p53 and p16INK4a. Nevertheless, our results did not identify any main pathways. Determining whether the pathways are interdependent or independent requires further investigation.

We conclude that MSC replicative senescence can be efficiently prevented using human umbilical cord-derived WJE. Umbilical cords are normally discarded as medical waste so no ethical concerns are involved in obtaining WJE which can easily be prepared in large quantities. Clinical requirements might be satisfied if sufficient amounts of MSCs can be prepared by culturing on WJE.
